# Comparison of transcripts in *Phalaenopsis bellina *and *Phalaenopsis equestris *(Orchidaceae) flowers to deduce monoterpene biosynthesis pathway

**DOI:** 10.1186/1471-2229-6-14

**Published:** 2006-07-13

**Authors:** Yu-Yun Hsiao, Wen-Chieh Tsai, Chang-Sheng Kuoh, Tian-Hsiang Huang, Hei-Chia Wang, Tian-Shung Wu, Yann-Lii Leu, Wen-Huei Chen, Hong-Hwa Chen

**Affiliations:** 1 Department of Life Sciences, National Cheng-Kung University, Tainan 701, Taiwan; 2 Institue of Information Management, National Cheng Kung University, Tainan 701, Taiwan; 3 Department of Chemistry, National Cheng Kung University, Tainan 701, Taiwan; 4 Graduate Institute of Natural Products, Chang Gung University, Taoyuan 333, Taiwan; 5 Department of Life Sciences, National University of Kaohsiung, Kaohsiung 811, Taiwan; 6 Institute of Biotechnology, National Cheng Kung University, Tainan 701, Taiwan; 7 Department of Biological Science and Technology, Chung-Hwa College of Medical Technology, Tainan Hsien 717, Taiwan

## Abstract

**Background:**

Floral scent is one of the important strategies for ensuring fertilization and for determining seed or fruit set. Research on plant scents has hampered mainly by the invisibility of this character, its dynamic nature, and complex mixtures of components that are present in very small quantities. Most progress in scent research, as in other areas of plant biology, has come from the use of molecular and biochemical techniques. Although volatile components have been identified in several orchid species, the biosynthetic pathways of orchid flower fragrance are far from understood. We investigated how flower fragrance was generated in certain *Phalaenopsis *orchids by determining the chemical components of the floral scent, identifying floral expressed-sequence-tags (ESTs), and deducing the pathways of floral scent biosynthesis in *Phalaneopsis bellina *by bioinformatics analysis.

**Results:**

The main chemical components in the *P. bellina *flower were shown by gas chromatography-mass spectrometry to be monoterpenoids, benzenoids and phenylpropanoids. The set of floral scent producing enzymes in the biosynthetic pathway from glyceraldehyde-3-phosphate (G3P) to geraniol and linalool were recognized through data mining of the *P. bellina *floral EST database (dbEST). Transcripts preferentially expressed in *P. bellina *were distinguished by comparing the scent floral dbEST to that of a scentless species, *P. equestris*, and included those encoding lipoxygenase, epimerase, diacylglycerol kinase and geranyl diphosphate synthase. In addition, EST filtering results showed that transcripts encoding signal transduction and Myb transcription factors and methyltransferase, in addition to those for scent biosynthesis, were detected by *in silico *hybridization of the *P. bellina *unigene database against those of the scentless species, rice and *Arabidopsis*. Altogether, we pinpointed 66% of the biosynthetic steps from G3P to geraniol, linalool and their derivatives.

**Conclusion:**

This systems biology program combined chemical analysis, genomics and bioinformatics to elucidate the scent biosynthesis pathway and identify the relevant genes. It integrates the forward and reverse genetic approaches to knowledge discovery by which researchers can study non-model plants.

## Background

Floral scent is a key modulating factor in plant-insect interactions and thus plays a central role in successful pollination. Closely related plant species that rely on different insects for pollination produce different odors [1,2]. The floral scent is of paramount importance to plant reproduction and evolution [3]. *Orchidaceae *is one of the largest monocotyledon families, containing more than 25,000 species. In orchids, large quantities of pollen are formed in masses spread by animals (bees, moths, flies and birds) and the floral scents serve as attractants for species-specific pollinators [4]. These pollinators have played a major role in orchid evolution. The range of odors produced by orchids is enormous, providing an inexhaustible basis for specificity. Such diversity is advantageous in the evolution of an obviously successful family.

The floral scent is determined by a specific complex mixture of volatile low-molecular-mass molecules dominated by monoterpenoid, sesquiterpenoid, phenylpropanoid and benzenoid compounds and fatty acid derivatives [5]. Although the complete pathways leading to the final products have not been characterized, common modifications such as hydroxylation, acetylation and methylation have been described [6]. These terpenes are synthesized from isopentenyl diphosphate by different mono and sesquiterpene synthases [7] and via two alternative pathways: the mevalonate pathway from acetyl-CoA, and the methylerythritol phosphate pathway from pyruvate and glyceraldehyde-3-phosphate, G3P [8]. Recently, the chemical structures of many floral scent compounds have been determined and several investigations into their biosynthesis have been described, [9]. The genes encoding enzymes such as linalool synthase, benzylalcohol acetyltransferase and 2 methyltransferases, involved in the biosynthesis of *Clarkia breweri *scent volatiles, have been isolated and characterized [10]. Similar results were obtained on a methyltransferase that catalyzes methyl benzoate formation in the petals of the snapdragon, *Antirrhinum majus *[11], and sesquiterpene synthase in rose petals [9]. In general, expression of these genes is highest in petals and is restricted to the epidermal cell layers of floral tissues [10].

Recently, expressed sequence tag (EST) has appeared as a powerful tool for genomics research. ESTs have developed into a useful way of discovering the genes for metabolic pathway enzymes in general [12-14]. These high-throughput technologies have therefore helped in identifying new fragrance genes in vegetative tissues and flowers, including three new genes from the deoxyxylulose-5-phosphate (DXP) pathway of isoprenoid biosynthesis in peppermint, *Mentha × Piperita *[15,16], phenylpropene metabolism in sweet basil, *Ocimum basilicum *[17], diterpene synthesis in *Stevia rivaudiana of Asteraceae *[18], terpene synthase in *Arabidopsis *[19,20], and rose scent-related genes [18]. However, the metabolic pathways of volatiles in monocotyledons have not been studied exhaustively and may differ from those in dicotyledons.

*Orchidaceae *have sophisticated flower morphologies including two whorls of perianth segments, three sepals and three petals, one of which is highly evolved as a labellum (lip) and serves as a landing platform for pollinators. The *Orchidaceae *species pollinated by bees, wasps and bumble bees cover a wide range of scents, from rosy- and ionone-floral to aromatic- and spicy-floral. Many volatile components have been identified in *Orchidaceae *flowers from the American tropics, African tropics, Australian tropics and parts of Europe [21]. However, the biosynthetic pathways of orchid flower fragrance are not well understood. Little is known about the enzymes and genes controlling scent production in monocotyledons such as orchids, and the problem of understanding the molecular mechanisms involved is perhaps insurmountable without genomics.

In this report, we describe the combined use of chemical analysis, genomics and bioinformatics to uncover the scent biosynthesis pathway and the relevant genes. Volatiles from the flower of a scented *Phalaenopsis *species, *P. bellina*, were compared with those from a scentless species by detailed chemical analysis and by identifying secondary metabolism-related genes using EST. We also generated scent-related ESTs by data mining as well as by EST filtering of the *P. bellina *unigenes database against those of scentless *Phalaenopsis*, rice and *Arabidopsis *to identify relevant genes. We characterized genes encoding enzymes in the biosynthetic pathway from pyruvate and G3P to geraniol, linalool and their derivatives. Expression of the scent-related genes was confirmed using RNA blot hybridization. This is the first attempt to deduce the scent biosynthesis pathway in *Phalaenopsis *orchids through systems biology, and the results give biochemical insights into monoterpene biosynthesis in these species.

## Results

### Major classes of volatiles emitted from *P. bellina *and *P. equestris *flowers

**Figure 1:**
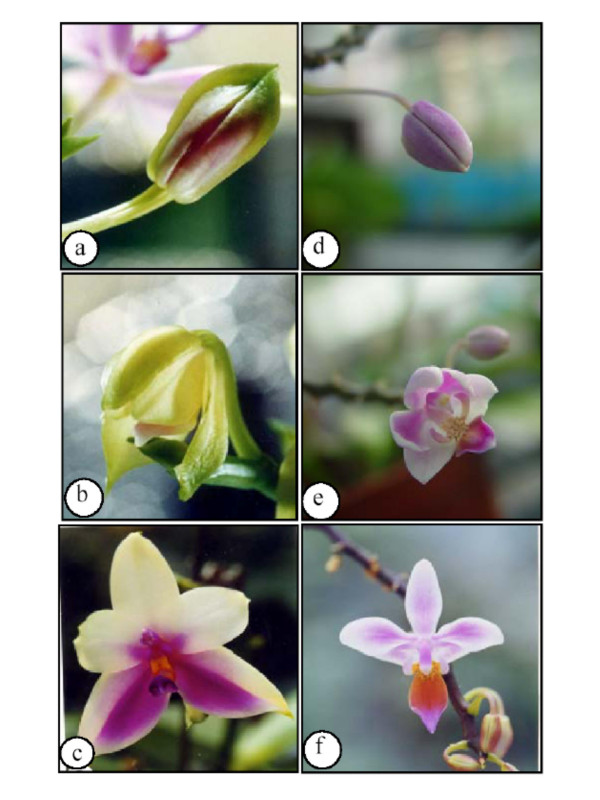
***P. bellina *(a-c) and *P. equestris *(d-f) flowers at different developmental stages**. a, d: Day 3 pre-anthesis; b, e: Flowering day; c, f: Day 7 post-anthesis.

Taxonomically, the genus *Phalaenopsis *belongs to the family *Orchidaceae*, sub-family Epidendroideae, tribe *Vandeae *and subtribe *Aeridinae*. The genus *Phalaenopsis *is subdivided into five subgenera, namely *Proboscidioides*, *Aphyllae*, *Parishianae*, *Polychilos *and *Phalaenopsis*, and comprises approximately 66 species according to the latest classification of Christenson [22]. *P. bellina *(Figure 1a–1c), classified in subgenus *Polychilos*, is native to Malaysia and numerous commercial varieties have been bred because of its pleasant fragrance. It has no linesof scentless varieties, so *P. equestris *(Figure 1d–1f) was used for comparison. *P. equestris*, subgenus *Phalaenopsis*, is a scentless species native to Taiwan with a colorful perianth. The subgenera *Polychilos *and *Phalaenopsis *diverged more than 21 Mya [23].

All species of *Phalaenopsis *display a remarkably uniform diploid chromosome number of 38 (2n = 2x = 38); however, their chromosome sizes and centrosome positions vary considerably [24,25]. Although the *P. bellina *karyotype shows large chromosomes with a 2C value of 15.03 pg and that of *P. equestris *shows small chromosomes with a 2C value of 3.37 pg [24,25], the two species can be crossed to yield progeny (personal communication from research members at Taiwan Sugar Research Institute).

Qualitative and quantitative analyses of volatile compounds from *P. bellina *flowers were performed using GC-MS. Monoterpenoids, phenylpropanoids, benzenoids and fatty acid derivatives were detected (Table 1). Monoterpenoids including geraniol, linalool and their derivatives (Table 1; see Additional file 1) accounted for more than 80% of the total volatiles collected from the *P. bellina *flowers. They included geraniol, nerol, 2,6-dimethyl-octa-3,7-diene-2,6-diol, 2,6-dimethyl-octa-1,7-diene-3,6-diol, 3,7-dimethyl-2,6-octadienal, geranic acid and 2,6-dimethyl-octa-2,6-diene-1,8-diol (see Additional file 1). In contrast, no monoterpenoid derivatives were emitted in the scentless *P. equestris *flowers; fatty acid derivatives, phenylpropanoids, and benzenoids were the major volatiles (Table 1; see Additional file 1). These compounds are barely detectable by the human nose.

### Characterization of *P. bellina *floral dbEST

Previously, we found that the scent-emitting structures of *P. bellina *were mostly in its perianth but not in its column (data not shown). The column of an orchid flower is a specialized organ comprising fused stamen and carpel, and many genes are involved in regulating its development. To reduce the complexity, we constructed a cDNA library from *P. bellina *flower buds with the columns removed immediately before blooming.

**Figure 2:**
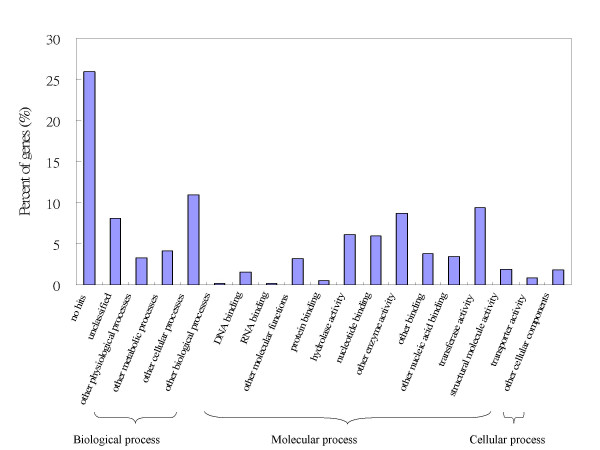
Functional classification of *P. bellina *flower bud dbEST.

A total of 2,359 individual 5'-ESTs was retained for the *P. bellina *flower bud with an average length of 916 bp, and the confidence level of the sequences called using PHRED was 22.8 per base. After 247 cDNA clones were analyzed by restriction enzyme digestion, the average insert size was 1.2 ± 0.32 kb. For better quality annotation, the ESTs were first refined and then the related genes were assembled as unigenes. The assembly program, Sequencher V. 4.1.2, was used to organize the redundant ESTs into unigenes of overlapping contigs at a stringency of 95% identity, with a minimum of 40 bases overlap. This process generated 1,187 unigenes: 499 contigs and 688 singletons. In the *P. bellina *floral dbEST, 1,748 (74.1%) of the 2,359 ESTs showed similarities to known sequences in the Uniprot database. The sequences in the flower bud dbEST were functionally characterized using a Gene Ontology (GO) scheme [26]. Details of the gene species included in each group are given in Figure 2. GO allowed 45.6% of the total ESTs to be placed in the molecular function category, 18.5% in the biological process category and 1.8% in the cellular component category. The remaining 34% either showed insufficient similarities to any proteins (no hits, 25.9%) or hit proteins without a GO identifier (unclassified, 8.1%). Among the molecular functions, the categories most highly represented were the transferases (9.4%), other enzymes excluding hydrolases, kinases and transferases (8.7%), and hydrolases (5.9%). Among the biological processes, the largest proportion (10.9%) of functionally assigned ESTs fell into cellular processes excluding signal transduction, cell organization and biogenesis and transport; other metabolic processes (excluding protein metabolism, DNA metabolism, RNA metabolism, electron transport, energy pathways and transcription) accounted for 4.1%. Together, these two classes of molecular functions and biological processes accounted for 64.1% of the assignable ESTs (Figure 2).

### Identification of scent biosynthesis pathway using the Pathway and Literature finder (PaL)

Analysis of the volatiles showed that monoterpenoids, including geraniol, linalool and their derivatives, are major compounds of *P. bellina *flower scent (Table 1), so it is reasonable to speculate that monoterpenoids are biosynthesized in these flowers. To identify candidate genes in the DXP-geraniol-linalool pathway of *Phalaenopsis*, we developed a Pathway and Literature finder system [27]. Five deoxyxylulose-5-phosphate synthase (DXPS) and one deoxyxylulose-5-phosphate reductase (DXPR) related ESTs were mapped to the methylerythritol phosphate (MEP) pathway, which was included in the Biosynthesis of Steroid pathway in KEGG database. No ESTs were mapped to the Terpenoid Biosynthesis or Monoterpenoid Biosynthesis pathways in KEGG. Possible reasons why only two types of ESTs were identified corresponding to the MEP pathway might include sequence diversification in different species and incompleteness of the KEGG collection of pathways. To extract further ESTs related to the DXP-geraniol-linalool pathway, EST annotations, the volatile components identified by GC-MS and the relevant PubMed literature were combined.

In the PaL system, we used 'plant terpene biosynthesis pathway and linalool' or 'plant linalool compound and scent' or 'plant geraniol' as background set keywords to collect literature from PubMed, and 'geranyl diphospate synthase' as functional keywords to search EST annotations. The program then connected the chosen keywords, ESTs and literature (see Additional file 3). Other sets of keywords were also applied to repeat the process (see Additional file 3).

On this basis, we elucidated the floral scent biosynthesis pathway in *P. bellina *further in accordance with the GC-MS data on geraniol, 2,6-dimethyl-octa-2,6-dien-1,8-diol and citronellol (Figure 4). We identified major steps in the pathway from pyruvate and G3P to geraniol, linalool and their derivatives (Figure 4). These included genes encoding deoxyxylulose-5-phosphate synthase (DXPS), deoxyxylulose-5-phosphate reductase (DXPR), 4-diphosphocytidyl-2-C-methyl-D-erythritol-2-phosphate cyclase (DMEC), EPI, GDPS and cytochrome P450. The biosynthetic pathway for linalool and its derivatives, including linalool oxide, was also deduced (Figure 4).

### ESTs differentially expressed between the floral dbESTs of *P. bellina *and *P. equestris*

Comparisons of the most abundant ESTs by calculating the enrichment factor (defined below) for different transcripts in the floral dbESTs of *P. bellina *and *P. equestris *[28,29] facilitate the provisional identification of scent metabolism genes. The enrichment factor was obtained by dividing the proportion of a certain transcript in the scented species by that in the scentless species. Statistical significance was determined using the simple Monte Carlo Test (α ≤ 0.05). Geranyl diphosphate synthase (GDPS), EPI, LOX and diacylglycerol kinase (DAK) showed 16.6, 10.0, 9.9 and 9.5-fold enrichments, respectively (Table 2). GDPS was significantly differentially expressed in the scented species (0.30% in *P. bellina *vs. 0.02% in *P. equestris*, Table 3). GDPS participates in the biosynthesis of monoterpenes in plastids [30] primarily by supplying the essential precursor, suggesting that *P. bellina *may produce scent molecules in the plastids rather than the cytoplasm. Among the ESTs of the *P. bellina *flower bud, EPI (1.61% in *P. bellina *vs. 0.16% in *P. equestris*, Table 2) is a multi-functional protein with hydratase, dehydrogenase, epimerase and isomerase activities involved in fatty acid beta-oxidation in plant peroxisomes and glyoxysomes [31,32]. DAK, enriched in the scented species (0.34% in *P. bellina *vs. 0.04% in *P. equestris*, Table 2), converts diacylglycerol to phosphatidic acid, which has been shown to accumulate rapidly in plant cells in response to stimuli and may function as a signal molecule [33-35]. If so, DAK may serve as a signal molecule for day-fragrance in *P. bellina *rather than being involved in scent biosynthesis directly.

In addition, expansin showed a 4.2-fold higher abundance in the scented species (0.68% in *P. bellina *vs. 0.16% in *P. equestris*, Table 2). Expansin loosens plant cell walls and thereby stimulates cell enlargement [36]. In orchids, expansin may burst the cell walls in the epithelium to release scent components. Indeed, transmission electron microscopy shows that bulges extend from the outer epidermis cell walls during flowering of *P. bellina *(unpublished results). Interestingly, our results show that peroxidase (1.36% in *P. bellina *vs. 0.39% in *P. equestris*, a 3.5-fold enrichment) might be related to scent metabolism.

More than 2-fold increases of O-methyltransferase transcripts (0.78% in *P. bellina *vs. 0.30% in *P. equestris*, a 2.6-fold enrichment) and cytochrome P450 transcripts (0.97% in *P. bellina *vs. 0.48% in *P. equestris*, a 2-fold enrichment) were also observed in *P. bellina *flowers. Previous research has shown that O-methyltransferase is involved in many reactions in volatile biosynthesis [37]. O-methyltransferase methylates a wide range of substrates such as catechols, phenylpropanoids, orcinol and isoquinoline, all of which are involved in modifying scent molecules upon induction [38]. Cytochrome P450 can act as a hydroperoxide lyase and catalyze the cleavage of lipoxygenase products (fatty acid hydroperoxides), forming omega-oxoacids and volatile C6- and C9-aldehydes and alcohols [39]. However, in some cases, fewer enzymes than expected are required to synthesize the various hydroxylated structures, such as cytochrome P450 mono-oxygenase in *Mentha *species [40] and geraniol 10-hydroxylase in *Catharanthus roseus *[41]. Molybdopterin biosynthesis cofactors (0.21% in *P. bellina *vs. 0.0% in *P. equestris*, Table 1) are involved in various types of oxidative metabolism, including nitrogen metabolism and phytohormone biosynthesis [42].

### Lipoxygenase (LOX) EST was the most abundant transcript in the *P. bellina *flower bud dbEST

It has been proposed that LOX may be involved in plant growth and development; biosynthesis of regulatory molecules such as jasmonic acid and traumatin; and biosynthesis of volatile compounds such as hexanal, hexenal and hexenol, which are involved in flavor, insect attraction and defense [43,44]. LOX enzymes can be grouped into two types: 9-LOX, which specifically forms 9- hydroperoxy derivatives of fatty acids (9-HPOs), and 13-LOX, which specifically forms 13-HPOs [44]. Interestingly, we discovered that three different kinds of 9-LOX-like ESTs (see Additional file 2) accounted for 2.12% of the *P. bellina *flower dbEST (Table 2). One of them (*PLOX3*) was specific to the *P. bellina *flower. However, no 13-LOX-like ESTs were observed in such flowers. In contrast, only 0.21% of the floral dbEST of *P. equestris *encoded LOX, and both 9-LOX and 13-LOX were detected (Tables 2 and see Additional file 2). In addition, four cytochrome P450 enzymes of the CYP74 subfamily-related ESTs represented as one gene were mined in the *P. bellina *flower dbEST. This contig comprised 1,115 nucleotides and showed 75% similarity at the amino acid level to rice allene oxide synthase (OsAOS, accession no: AAL17675) and 72% similarity to *Cucumis melo *9-hydroperoxide lyase (CmHPL, accession no: AAK54282) [45,46] The high percentage of 9-LOX-related ESTs, and the existence of ESTs associated with the AOS or HPL pathway, together suggest that the 9-LOX pathway for linoleic or linolenic acid metabolism is executed in the *P. bellina *flower.

### Identification of scent-related genes by EST filtering

By adopting previous approaches, we could have identified those scent-related genes that have already been reported in the literature. However, novel scent-related genes that have not been reported, or their sequences are too divergent from known ones, would not be identified by using the PaL finder. In addition, genes with low expression levels would not easily be identified by enrichment factor analysis. To detect still more scent-related genes, we performed EST filtering using less stringent conditions to remove genes homologous between *P. bellina *and *P. equestris*. The 1,187 *P. bellina *unigenes were filtered against the 3,555 floral unigenes of *P. equestris *using TBLASTX at an E-value < 10^-5^. The ESTs left over after filtering with the minimum acceptable threshold (E-value < 10^-5^) could plausibly represent non-homologous transcripts in *P. bellina*.

The results showed that 820 unigenes of *P. bellina *did not match the *P. equestris *unigene database (Table 3). The EST filtering results revealed genes involved in the biosynthetic pathway from pyruvate and G3P to linalool, geraniol and their derivatives in floral unigenes of *P. bellina*, consistent with the chemical analysis results showing that no linalool and geraniol were emitted from the floral organs of *P. equestris*. However, the filtered results may not account only for fragrance genes; some filtered unigenes may be involved in morphogenetic networks. Interestingly, *P. bellina *(colored from red to orange in petal and lip) and *P. equestris *(red petal, orange lip) have similar color spectra, so EST filtering eliminated the genes involved in the biosynthesis pathways for flower colors and reduced the complexity of analysis. Indeed, the fact that no anthocyanin biosynthesis genes were identified in the EST filtering results is evidence for the efficacy of EST filtering.

Further EST filtering by use of TBLASTX against the collected floral unigenes of rice and *Arabidopsis*, which both lack fragrance, removed the genes involved in morphogenesis. An E-value < 10^-7 ^was applied for the TBLASTX program, and the matched results with fewer ribosomal proteins and housekeeping genes were accepted. Unigenes with significant BLAST search results were classified as homologous genes among rice, *Arabidopsis *and *P. bellina*. Altogether, 365 unigenes remained unmatched and included fewer housekeeping genes at E-value < 10^-7^, and thus were defined as *P. bellina *scent-related genes (Table 3).

A total of 330 unigenes common to the above two EST filtering results constituted a more refined set of scent-related genes in *P. bellina *(Table 3). These transcripts included genes for NADPHDH, EPI, molybdopterin biosynthesis cofactors and GDPS (Table 3). Concomitantly, EPI and GDPS were among the highly expressed transcripts, and molybdopterin biosynthesis cofactors were only expressed in the *P. bellina *flower buds (Table 2). Our results also suggest that NADPHDH and cytochrome P450 are required for the formation of linalool and geraniol derivatives (Figure 4). From the chemical and bioinformatic analyses, we deduced a monoterpene biosynthesis pathway of 15 steps in the *P. bellina *flower, leading from G3P to geraniol, linalool and their derivatives. ESTs corresponding to 10 of these steps (66 %) were identified (Figure 4).

Transcripts encoding signal transduction factors such as sensor proteins, membrane proteins and mitogen-activated protein kinase were also identified (Table 3), suggesting that scent emission may be related to stimuli that elicit a series of signal transduction processes leading to gene expression and scent production. We also detected ferrochelatase, as well as Myb family protein (Table 3), which has been shown to regulate the biosynthesis of petunia flower fragrance [47]. However, we could not rule out the possibility that the ESTs identified by enrichment factor analysis were merely the results of incompleteness and bias in the dataset used in this analysis.

### Confirmation of scent-related genes by RNA blot hybridization

Differential expression of several of the identified scent-related genes was confirmed by RNA blot hybridization. *PEPI3 *was highly expressed in the scented species but rarely expressed in the scentless species (Figure 5). Likewise, *PLOX1, PGDPS *and *PNADPHDH1 *were expressed in the scent species. The same blot was hybridized to a probe encoding a ribosomal RNA indicating that each individual lane had been loaded with the same amount of RNA (Figure 5). These results were consistent with the bioinformatics analysis of floral ESTs preferentially expressed in the scent species.

## Discussion

In this report, we developed a PaL system to facilitate the identification of literature and pathway information related to certain ESTs. Unlike PubMed, which can only provide keyword searches without identifying the papers really related to ESTs of interest to the user, PaL also provides an easier search platform for demonstrating which of the sequences in hand is engaged in a certain KEGG pathway. In PaL, BLAST is used for alignment in batch (BLASTALL) and its alignment results are parsed to extract such information. Thus, the PaL system covers the BLAST function. We drew the candidate scent biosynthesis pathway manually. In addition, we recognized enriched transcripts by comparing the floral unigene databases for scent and scentless species, and by EST filtering to identify other genes for scent biosynthesis and scent modification candidates. Systematic collection of ESTs and retrieval of related research documents from public databases were highly rewarding strategies for studying these non-model plants, particularly since genomic information is limited in orchids. Efficient connection of the biological components with related literature by computational methods was a useful strategy for deducing the metabolic pathways.

In this study, we were able to identify genes, including those for DXPS, DXPR, DMEC, GDPS, EPI, NADPHDH and cytochrome P450, all involved in the DXP-geraniol-linalool pathway, by combining EST data mining with metabolic profiling and analysis of volatiles and EST filtering. Although comparison with scentless plant material would in principle be optimal if isogenic scentless *P. bellina *lines could be used, no such variants yet exist. The flowers of *P. bellina *and *P. equestris *express similar sets of genes determining the nature of the organ even though they are phenotypically different. However, their final appearance, including scent, may be determined by a relatively small number of genes expressed in a cultivar-specific manner.

All monoterpenes are formed from GDP, which is synthesized from dimethylallyl diphosphate and isopentenyl diphosphate [48]. The high expression level of GDPS in *P. bellina *flowers suggests that scent biosynthesis in this species is predominately due to production of geraniol and linalool from DXP. Thus, EST data mining and EST filtering were informative in identifying genes among different dbESTs from different sources and may be applicable for comparative genomics.

Monoterpene synthase genes have been identified in both floral and vegetative organs of several angiosperms and gymnosperms [19,49] Of special interest in this respect are studies of terpene synthases, a large class of enzymes that appear to be responsible for most of the structural variety of terpenes [50]. However, terpene synthase was not identified in *P. bellina *in this work. We have applied an HMM based method such as Interpro [51] to validate whether any such protein domains were in fact present in the dataset. Results showed that no any terpene synthase domains were present in the *P. bellina *floral EST database. The low sequence-relatedness among monoterpene synthases in angiosperms [49] might have added to the difficulty; thus far, few terpene synthase genes have been identified in monocotyledons. In addition, these genes may belong to families with high diversity in non-conserved regions [10]. A further attempt to identify them was conducted by *in silico *hybridization using known monoterpene synthase genes as electronic probes, but neither linalool synthase nor geraniol synthase genes were found. The pool in the *P. bellina *EST database may be insufficient, so that not all genes in the scent biosynthesis pathway – including monoterpene synthase genes – have been identified. Alternatively, there may be regulation of the scent biosynthesis at the precursor level, and the enzymes responsible for synthesis are not transcriptionally regulated. Previous studies have shown that linalool synthase levels and activities in *Clarkia breweri *remain high while linalool emission decreases, suggesting that regulation of terpenoid precursors occurs in *C. breweri *[10,52].

Both EPI and NADPHDH are multifunctional and are involved in β-oxidation and reduction reactions [53]. *PEPI3 *showed a distinct expression profile in the scented species and thus may be correlated with scent biosynthesis via the geraniol-linalool pathway. Altogether, our results suggest that EPI, cytochrome P450 and NADPHDH are related to monoterpenoid biosynthesis in orchids.

The role of the LOX pathway in plant-pathogen interactions and the importance of 13-LOXs and their product jasmonate in resistance against insects and pathogens have been analyzed in numerous pathosystems [54,55] The 9-LOX pathway generates a group of metabolites that are structurally related to, but distinct from, those derived from the 13-LOX pathway [55]. In our results, a much lower level of fatty acid derivatives was observed in *P. bellina *flowers than in *P. equestris *flowers (3.3 ± 1.8 vs. 330.5 ± 35.0 ng/flower/h; Table 1). Since no 13-LOX pathway genes were expressed in *P. bellina*, its fatty acid derivatives were produced mostly by the 9-LOX pathway. However, there was a discrepancy in that 9-LOX ESTs were the most abundant ESTs in *P. bellina *flowers, but fatty acid derivatives were at very low levels in its volatiles. These results suggest that fatty acid derivatives produced by the 9-LOX pathway in *P. bellina *flowers are mostly not volatiles. Recently, 9-hydroxy-10-oxo-12(Z), 15(Z)-octadecandienoic acid (KODA) biosynthesized by 9-LOX and AOS were shown to have flower-inducing activity in *Lemna *[56]. Although the biological function of the high levels of *9-LOX *expression in *P. bellina *flowers is unclear, the expression of *PbLOX *may control the synthesis of some signal for flower scent formation or emission. In addition, many lipid bodies were observed in the petal epidermis of *P. bellina *by transmission electron microscopy (data not shown), suggesting that the 9-LOXs are involved in converting storage lipids into substrates for further oxidation to provide energy for scent emission. It will be important to demonstrate products of the 9-LOX pathway *in vivo *and functionally characterize genes that encode 9-LOX and other enzymes involved in this pathway in order to understand their relationships to floral scent formation and emission in *P. bellina*.

Despite the popularity of *Phalaenopsis *species in cultivation, very little is known about their ecology and distribution in nature. So far, there have been no reports indicating the kind of insects that visit *P. bellina *or *P. equestris *in the wild. Flowers of *Phalaenopsis *spp., with its colorful perianth, are pollinated by bees and produce fragrances during the day [22]. Bumble bees are primarily attracted from a distance to visual stimuli, whereas landing depends upon both visual and olfactory cues [57]. The complex fragrances and vivid colors in *P. bellina *suggest that this species probably attracts pollinators by both olfactory and visual stimuli. In contrast, the strictly visual strategy used by *P. equestris *to attract pollination vectors is the presentation of the colored perianth. Although *P. equestris *has small flowers (1.0–1.2 cm in diameter), it produces many more flowers in a peduncle compared to *P. bellina*, which produces 1 to 3 larger flowers (5–6 cm in diameter) per plant but with fragrance. It seems logical that the evolution of *Orchidaceae*, a family that was probably already highly "pollinator-oriented" at its inception, further augmented the complexity of the pollination mechanisms [57]. The emission of volatiles from *P. bellina *flowers may have evolutionary significance and thus increase reproductive fitness in orchids.

## Conclusion

In this work we have shown how EST research can be usefully applied to the construction of a putative scent metabolism pathway in *P. bellina *and the identification of the genes encoding the enzymes involved in this pathway. For a non-model plant with a very large genome (1.5 ~ 8.1 × 10^9 ^bp for *Phalaenopsis *spp. [25]), which cannot easily be accessed for whole genome sequencing, EST analysis of its transcriptome profile becomes a very efficient and informative tool. A combination of volatile analysis, EST database mining with the PaL finder and EST filtering can be applied to deduce the scent biosynthesis pathway in *P. bellina *and to identify scent-related genes.

## Methods

### Plant materials

The development of *P. bellina *flowers is divided into 3 stages. At stage 1 (day 3 pre-anthesis), the flower bud is closed, the petals are green and no fragrance is emitted (Figure 1a). At stage 2 (the flowering day), the petals and sepals blossom slightly but still without fragrance (Figure 1b). At stage 3 (day 7 post-anthesis), *P. bellina *flowers bloom and have a strong fragrance (Figure 1c). In contrast, *P. equestris *flowers are scentless (Figure 1d–1f). All plant materials were cultivated in the greenhouse at the Taiwan Sugar Research Institute (TSRI) in southern Taiwan. The growth conditions were: temperature 30°C/25°C, relative humidity 84% and photosynthetic photon flux density 90 μmol m^-2 ^s^-1^.

### Headspace scent chemical collection and chromatographic analysis

*P. bellina *and *P. equestris *flowers produce fragrance during the day. Volatiles trapped from day 5 to day 10 post-anthesis flowers (35 *P. bellina *and 24 *P. equestris*) were collected using dynamic headspace sampling systems [5], with air pumped from the chamber through activated charcoal traps (1.5 mg) at 400 ml/min for 35 h (from 9 am to 4 pm for 5 days). The volatiles were eluted with 1 ml hexane and evaporated to 0.5 ml. Trapped floral scent compounds were analyzed by gas chromatography-mass spectrometry (GC-MS; QP2010, SHIMADXU, Shimadzu Co, Tokyo, Japan). We used an INNOWAX column (60 cm, 0.32 mm, 0.25 μm phase thickness) and the oven was programmed from 40°C to 230°C (held for 5 min) at 5°C/min increments. The pressure of the helium inlet was set at 75.2 kPa, with a linear velocity of 34.6 cm/s (split flow 8.3 μl/min). The injector temperature was kept at 240°C, with the injected volume set to 1 μl and the electron energy to 70 eV. Mass spectra and reconstructed chromatograms were obtained by automatic scanning of the samples in the mass range *m/z *20–500 Da. Peaks on mass chromatograms with characteristic fragments were checked for homogeneity.

The identities of all compounds were determined by comparing retention times and mass fragmentation patterns with the NIST98 (US Environmental Protection Agency, 1998) and NIST02 (SHIMADXU, Shimadzu Co, Tokyo, Japan) databases. For quantitative analyses, 10 μg/ml ethyl myristate was used as an internal standard [36].

### Construction of cDNA library

Total RNA samples were extracted from *P. bellina *flower buds (column removed) by the guanidium thiocyanate method [58]. Poly(A) mRNA was prepared with a Poly(A) Quick RNA Isolation kit (Stratagene, La Jolla, CA). The cDNA library was constructed using a commercial kit following the manufacturer's instructions (Stratagene). The cDNAs synthesized were directionally cloned into a ZAPII vector. cDNA phage clones were excised using the EX Assistant helper phage system (Stratagene), and a pBluescript SK+ plasmid was recovered. A floral EST database established previously from the flower buds of the scentless species *P. equetris *[21,24] was used for comparison.

### DNA sequencing and analysis

Plasmid DNAs were purified from overnight cultures with a miniprep kit (Viogene, Taipei, Taiwan). Sequencing reactions were carried out from the 5' end using an automated sequencer (ABI PRISMTM 377 DNA Sequencer, Perkin-Elmer, Boston, MA) with a T3 primer 5'-AATTAACCCTCACTAAAGGG-3'. Sequence data were analyzed with Sequencher V. 4.1.2 (Gene Codes Corp., Ann Arbor, MI) to remove vector, poly(A), adaptor and ambiguous sequences. The *P. bellina *flower bud EST sequences have been submitted to the EST database with the accession numbers CK857580-CK859399 and CO742089-CO742627.

The PHRED program was employed for base calling and sequence quality assessment. The assembly program, Sequencher V. 4.1.2, was then used to organize the redundant ESTs into unigenes of overlapping contigs at a stringency of 95% identity with a minimum 40 bases of overlap. All sequences were searched and checked for similarities to sequences in Uniprot [59] using the BLASTX tool [60]. The GO Slim Classification for Plants, developed at TAIR [61] was used to characterize the ESTs functionally. The GO identifier of the best hit (with a cutoff of 1e-5) was attributed to the sequence. This step allowed putative functions to be assigned on the basis of the classification proposed by GO.

Floral transcripts differentially expressed between the scented and scentless species were represented as percentages of ESTs in each floral bud cDNA library (Table 2). The simple Monte Carlo Test [62] was used to assess the statistical significance of the heterogeneity of distribution between the two EST databases. Each EST sequence in the two databases was randomly shuffled 1000 times. The frequencies of corresponding ESTs were calculated in the simulated databases. This procedure was repeated 19 times.

### Pathway and Literature (PaL) finder: a system for speeding up pathway finding

We developed a system, Pathway and Literature (PaL) finder, to map ESTs to metabolic pathways, and correlated the EST annotations and metabolic components to the related literature.

**Figure 3:**
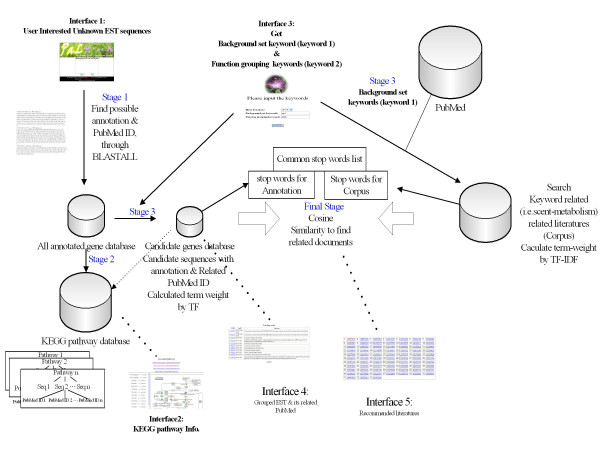
The flowchart of PaL finder.

**Figure 4:**
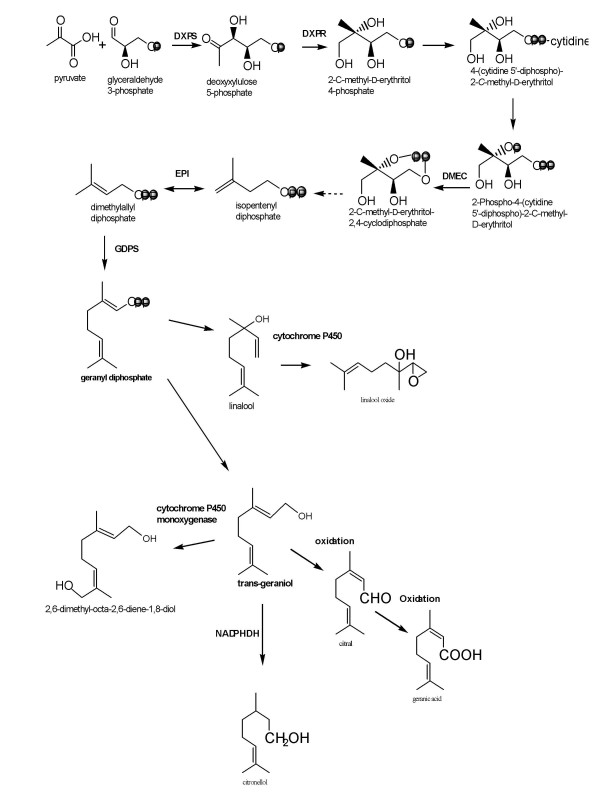
**Putative metabolic pathway from pyruvate and glyceraldehyde-3-phosphate to secent synthesis, and its related enzymes, in *P. bellina***. DXPS: deoxyxylulose-5-phosphate synthase; DXPR: deoxyxylulose-5-phosphate reductoisomerase; DMEC: 4-diphosphocytidyl-2-C-methyl-D-erythritol 2- phosphate cyclase; EPI: epimerase; GDPS: geranyl diphosphate synthase; NADPHDH: NADPH dehydrogenase.

**Figure 5:**
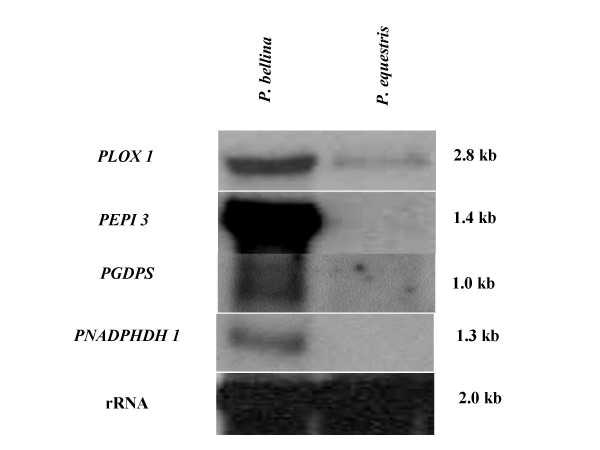
**RNA gel-blot analysis of mRNA transcripts at day 3 pre-anthesis**. RNA was extracted from day 3 pre-anthesis flower buds of *P. bellina *(lane 1) and *P. equestris *(lane 2) and analyzed for *PLOX1*, *PEPI3*, *PGTPS *and *PNADPH-dehydrogenase 1 *(*PNADPHDH1*) expression. The blots contained 10 μg of total RNA extracted from the different mature floral buds. The ribosomal RNA signals showed that the same amounts of total RNA were loaded on each individual lane.

Initially, the Pathway and Literature (PaL) finder was developed to speed up the process of finding pathways. The architecture of the PaL system is shown in Figure 3. Its outputs include possibly-involved pathways and related literature for sequences of interest to the user.

In this system, users are required at the outset to input a set of ESTs of interest in FASTA format. After receiving these data, the first stage of this system is to annotate those ESTs against the Arabidopsis proteome provided in the KEGG database through BLASTALL. This stage also collects directly related literature found from the BLAST search results. This stage can be time-consuming since BLASTALL needs a longer time to process all EST alignments.

At stage 2, sequences annotated in stage 1 are mapped to Arabidopsis metabolic pathways in KEGG. The pathway query environment for our VFCP database is demonstrated at taiwanorchid_1 [63].

At stage 3, a query environment is designed for users to collect a literature corpus and to narrow down the ESTs in which they are interested at taiwanorchid_2 [64]. This stage uses information retrieval techniques to rank the literature based on the correlation between publications and annotation of the ESTs of interest. Two sets of keywords are requested from the user. The first set, the background keyword set, normally has wider domain keywords (such as flower scent), which are used to collect domain-related literature for building a literature corpus. The other set (keywords 2) is used to filter the ESTs through data mining if their annotations contain the keywords of interest. Those ESTs filtered by using keywords 2 were in the same domain.

At final stage, the cosine similarity with vector space model (VSM) was used to find similarities between the grouped annotations and the literature in the corpus. In this stage, this program tokens, stems, waves stop word, and indexes the terms for both annotations and literature. Term frequency (TF, [65]) was used for the candidate gene database to calculate term weight and frequency with inverted document frequency (TF-IDF, [65]) in order to weight the corpus database. The output of this page is recommended literature, ordered according to correlation with the domain of interest to the user.

### EST filtering

To identify transcripts that are highly abundant in the scent species but in neither the scentless species nor rice and Arabidopsis flowers, we carried out 2 steps of EST filtering. First, the scent-related genes in the scent floral bud unigene database were filtered by *in silico *hybridization using the unigene database of the *P. equestris *flower bud as probes. The flower bud unigene database of *P. bellina *was filtered by TBASTX against the flower bud unigene database of *P. equestris *with an E-value < 10^-5^. The matched genes were counted as homologous genes between *P. bellina *and *P. equestris*, while the unmatched ones were counted as *P. bellina *specific and may be scent- or morphogenesis-related genes. The "no hits found" sequences were retrieved using an in-house program, nothitfound.pl, for further processing. Further EST filtering was performed against the collected (15,350) flower unigenes of rice and Arabidopsis, which were obtained from NCBI public databases. The TBLASTX program was carried out with an E-value < 10^-7^.

### RNA blot analysis

For northern blot hybridization, RNA was prepared from the flower buds of *P. bellina *and *P. equestris*. Total RNA samples of 10 μg were denatured with glyoxal, subjected to electrophoresis on a 1% agarose gel and transferred to nylon filters (Amersham Pharmacia Biotech, Piscataway, NJ). The RNA blots were hybridized with various probes for scent-related genes. The conserved domains of lipoxygenase (LOX), epimerase/dehydratase (EPI) and NADPH dehydrogenase (NADPHDH) were excluded from the probe-designing region to avoid cross-hybridization. The *PLOX1 *probe was generated with the primer pair 5'-CGCATCGGATGAGCTATATT-3' and 5'-GATGCAGAAACTTAGTACTGC. The *PEPI3 *probe was amplified with 5'-CACTTAAGCACATTTCTGGT-3' and 5'-TCGACAAATGATCTGGAGGA-3', and the *PNADPHDH1 *probe was amplified with 5'-GCTCCCAGTGTGCCTATGA TACC-3' and 5'-TCCCTCCCGCAATACG AATG-3'. The g*eranyl diphosphate synthase *(*PGDPS*) probe of *P. bellina *was generated with the primer pair 5'-GCGGTTAGGCGACTGCTT-3' and 5'-CAGAAT ACAATAATACATGAATATCACC-3'. Conditions for prehybridization and hybridization were as described by Tsai et al. [66]. For an internal control, the same blot was hybridized to a probe containing a partial genomic fragment coding for ribosomal RNA from *Phalaenopsis *(a gift from Dr. Y. Y. Kao, Institute of Molecular and Cellular Biology, National Taiwan University, Taipei, Taiwan).

## Authors' contributions

YY Hsiao designed the research pipeline, performed the library construction and sequence analysis and drafted the manuscript. WC Tsai and CS Kuoh carried out the programming. TH Huang and HC Wang developed the PaL finder and EST filtering methods. YL Leu and TS Wu helped in the analysis of flower volatiles. WH Chen suggested and offered the orchid materials. HH Chen conceived of and oversaw the research. All the authors read and approved the final manuscript.

## Supplementary Material

Additional File 1 Volatiles emitted from *P. bellina *and *P. equestris *flowers. Click here for file

Additional File 3 EST and related literature were linked by applying keywords to PaL finder system Click here for file

Additional File 2 Number of *Lipoxygenase *ESTs detected in *P. bellina *and in *P. equestris *flowers Click here for file

## Figures and Tables

**Table 1:** Major classes of volatiles emitted by *P. bellina *and *P. equestris*

	Amount (ng/flower/h)	
	
Class of Volatiles	*P. bellina *(scent)	*P. equestris *(scentless)
**Monoterpenes**	382.8 ± 16.1	ND^a^
Linalool	105.4 ± 15.3	ND^a^
Linalool derivaties	39.6 ± 20.0	ND^a^
*trans*-Geraniol	163.4 ± 1.6	ND^a^
Geraniol derivaties	34.5 ± 5.4	ND^a^
**Phenylpropanoid**	38.6 ± 17.1	109.0 ± 14.0
**Benzenoid**	40.2 ± 20.8	33.2 ± 7.0
**Fatty acid derivatives**	3.3 ± 1.8	330.5 ± 35.0

^a^ND: undetected

**Table 2:** Differential expressed transcripts between *P. bellina *(scent) and *P. equestris *(scentless) species in the floral cDNA libraries.

Putative functions	**Abundance in floral dbESTs**		**Enrichment factor**^**c**^
		
	***P. bellina *(%)**^**a**^	***P. equestris *(%)**^**b**^	
Geranyl diphosphate synthase	0.30	0.02	16.6
Epimerase/dehydratase	1.61	0.16	10.0
Lipoxygenase	2.12	0.21	9.9
Diacylglycerol kinase	0.34	0.04	9.5
Expansin	0.68	0.16	4.2
Peroxidase	1.36	0.39	3.5
Elongation factor	1.19	0.37	3.2
O-methyltransferase	0.78	0.30	2.6
Cytochrome P450	0.97	0.48	2.0
Heat shock protein	1.10	0.66	1.7
Class I chitinase	0.38	0.27	1.4
Molybdopterin biosynthesis cofactors	0.20	0.0	ND^d^

^a^Number of EST divided by 2359 ESTs of *P. bellina *floral dbESTs.

^b^Number of EST divided by 5593 ESTs of *P. equestris *floral dbESTs.

^c^Proportion (%) of ESTs in *P. bellina *an *P. equestris *floral bud libraries and the enrichment factor in the scent floral bud library (proportion of *P. bellina *flower).

^d^ND: undetermined

**Table 3:** EST filtering results using unigenes of *P. bellina *against those of *P. equestris*, Arabidopsis and rice.

**Annotation**	**Scent_Scentless(820)**^**a**^	**Scent_At+Os (365)**^**b**^	**Scent_Scentless_At+Os (330)**^**c**^
NADPH dehydrogenase	6	6	6
Molybdopterin biosynthesis cofactors	3	3	3
Membrane protein	6	5	3
Sensor protein	2	2	2
Geranyl diphosphate synthase	2	2	2
Ferrochelatase	1	1	1
Epimerase/dehydratase	1	1	1
Mitogen-activated protein kinases	1	1	1
Photosystem protein	1	1	1
Myb family protein	3	1	1
O-methyltransferase	1	1	1
vNo hits found	120	87	69
Hypothetical protein	81	49	43
Unknow protein	93	35	25

^a^The number of unigenes obtained in the EST filtering result at an E-value < e^-5^.

^b^The number of unigenes obtained in the EST filtering result at an E-value < e^-7^.

^c^The number of unigenes that were simultaneously present in previous two EST
