# Supplementary material for: Comparison of transcripts in Phalaenopsis bellina and Phalaenopsis equestris (Orchidaceae) flowers to deduce monoterpene biosynthesis pathway
Source: BMC Plant Biol. 2006 Jul 13;6:14. doi: 10.1186/1471-2229-6-14 (PMC1540424; doi:10.1186/1471-2229-6-14)
Supplement: Additional File 1 — Volatiles emitted from P. bellina and P. equestris flowers. [file 1471-2229-6-14-S1.pdf]

Additional file 1 - Volatiles emitted from *P. bellina* and *P. equestris* flowers

	P. bellina (scented)		*P. equestris* (scentless)	
**Compounds**	ng/flower/h	SE	ng/flower/h	SE
**Monoterpenes**				
Myrcene	12.0	7.1		
β-Pinene oxide	3.7	1.2		
Epoxy-terpenyl acetate	14.0	9.1		
Linalool	105.4	15.3		
3,7-Dimethyl-6-octen-1-ol	2.3	1.4		
3,7-Dimethyl-1,5,7-octatrien-3-ol	4.3	2.6		
2,6-Dimethyl-3,7-octadiene-2,6-diol	18.3	6.0		
2,6-Dimethyl-1,7-octadiene-3,6-diol	20.3	9.1		
Linalool oxide	3.4	2.4		
trans-Geraniol	163.4	1.6		
Nerol	6.0	1.9		
3,7-Dimethyl-2,6-octadienal	3.1	0.1		
Geranic acid	8.6	5.3		
2,6-Dimethyl-2,6-octadiene-1,8-diol	18.0	12.7		
**Total**	**382.8**	16.1		
**Phenylpranoids**				
3-Phenyl-2-propen-1-ol	3.7	2.6		
2,4-Bis(1,1-dimethylethyl)-phenol	4.0	1.7		
2,6-Bis(1,1-dimethylethyl)-4-methyl-phenol	18.9	11.1	104.4	13.0
2,6-Dimethoxy-4-(2-propenyl)-phenol	12.0	8.5	4.6	4.0
**Total**	**38.6**	17.1	**109.0**	14.0
**Benzenoids**				
1,2,3-Trimethoxy-5-(2-propenyl)-benzene	27.1	12.7		
3,5-Di-*tert*-butyl-4-hydroxybenzaldehyde	7.1	0.8		
3,5-Di-*tert*-butyl-4-hydroxybenzyl alcohol	6.0	4.2	6.5	2.1
3-Methylphenyl butanoic acid ester			10.0	1.3
2-Methylphenyl butanoic acid ester			10.5	2.0
Naphthalene			6.2	0.6
**Total**	**40.2**	20.8	**33.2**	7.0
**Fatty acid derivatives**				

2-Hexanol	0.7	0.5	40.2	2.0
4-Hydroxy-4-methyl-2-pentanone	2.6	1.5	8.6	1.1
3-Hexanone			17.3	1.9
5-Hexen-2-one			7.6	0.5
2-Nonanol			22.2	2.0
1-Methyl-cyclopentanol			18.5	5.1
4,6-Dimethyl-dodecane			19.8	4.6
Hexadecane			37.5	15.0
4-Methyl-dodecane			25.3	0.5
4-Methyl-2-propyl-1-pentanol			10.1	0.3
n-Nonylaldehyde			3.6	0.1
2-Butoxy-ethanol			3.3	0.1
2-Ethyl-1-hexanol			85.8	0.8
Heptadecanoic acid			30.7	1.0
Total	**3.3**	1.8	**330.5**	35.0
