# Supplementary material for: Comparison of transcripts in Phalaenopsis bellina and Phalaenopsis equestris (Orchidaceae) flowers to deduce monoterpene biosynthesis pathway
Source: BMC Plant Biol. 2006 Jul 13;6:14. doi: 10.1186/1471-2229-6-14 (PMC1540424; doi:10.1186/1471-2229-6-14)
Supplement: Additional File 3 — EST and related literature were linked by applying keywords to PaL finder system [file 1471-2229-6-14-S3.pdf]

Additional file 3 – EST and related literature were linked by applying keywords to PaL finder system[a].

**Functional keywords**	**geranyl diphosphate synthase**		
Background set keywords	Link EST	Literature found, PubMed ID	Related literature PubMed ID
plant terpene biosynthesis pathway and linalool	VFCP_Contig[0068]	14630967, 14756770, 15522848, 15618433, 16478276	14756770, 15522848, 15618433, 16478276
plant linalool compound and scent	VFCP_Contig[0068]	15031409, 16179482	15031409, 16179482
plant geraniol	VFCP_Contig[0068]	14657490, 15053766, 15184006, 15374632, 15474569, 15516500, 15666533, 15680985, 15680986, 15863882, 15890377, 16262708, 16525891	14657490, 15053766, 15184006, 15374632, 15474569, 15516500, 15890377, 16262708, 16525891

**Background set keywords**	**flower scent, flower terpene**		
Functional keywords	Link EST	Literature found, PubMed ID	Related literature PubMed ID
DXPS	VFCP-27A12, VFCP-27C3	15031409, 15122041, 15805488, 16233496	15031409, 15122041, 15805488, 16233496
DXPR	VFCP-33F10,		
lytB (DEMC)	VFCP-19E8	15031409, 16361520	15031409, 16361520

geranyl diphosphate synthase	VFCP_Contig[0068]	15031409, 15805488	15031409, 15805488
epimerase	VFCP_Contig[0087], VFCP_Contig[0148], VFCP_Contig[0240], VFCP_Contig[0270], VFCP_Contig[0289], VFCP_Contig[0313], VFCP_Contig[0345], VFCP_Contig[0346], VFCP-08C10, VFCP-08C4, VFCP-12C12, VFCP-15H12, VFCP-15H6, VFCP-16A2, VFCP-22H7, VFCP-25A2, VFCP-27G6	12969783, 14713699, 14742875, 14973286, 15031409, 15073212, 15120450, 15128936, 15128937, 15161963, 1517 3565, 15190082, 15604679, 15605174 15695465, 15700831, 15728344, 15816553, 15918888, 15964083, 15980191, 16098112, 16262715, 16400150, 16412086, 16421562	15031409, 14658517, 15201205, 15279302, 16361520
cytochrome P450	VFCP_Contig[0042], VFCP_Contig[0357], VFCP-08G12, VFCP-13A5, VFCP-14A7, VFCP-16A3, VFCP-19G6, VFCP-25F6, VFCP-25F9	15031409, 16361520, 16525870	15031409, 16361520, 16525870

[a]: The PaL system operates on-line. There are few difference in numbers of references found between searches at different times.
