# Supplementary material for: Comparison of transcripts in Phalaenopsis bellina and Phalaenopsis equestris (Orchidaceae) flowers to deduce monoterpene biosynthesis pathway
Source: BMC Plant Biol. 2006 Jul 13;6:14. doi: 10.1186/1471-2229-6-14 (PMC1540424; doi:10.1186/1471-2229-6-14)
Supplement: Additional File 2 — Number of Lipoxygenase ESTs detected in P. bellina and in P. equestris flowers [file 1471-2229-6-14-S2.pdf]

Additional file 2 – Number of *Lipoxygenase* ESTs detected in *P. bellina* and in *P. equestris* flowers

Gene	Class	*P. bellina*	*P. equestris*	Similarity (%)	E-value	Species
PLOX1	9-LOX	34	9	78	0	*Solanum tuberosum*
PLOX2	9-LOX	4	2	79	4.00E-77	*Nicotiana attenuata*
PLOX3	9-LOX	12	0	76	6.00E-43	*Lycopersicon esculentum*
PLOX4	13-LOX	0	7	68	4.00E-11	*Prunus dulcis*
